# Bacterial Infection Diagnosis and Antibiotic Prescription in 3 h as an Answer to Antibiotic Resistance: The Case of Urinary Tract Infections

**DOI:** 10.3390/antibiotics10101168

**Published:** 2021-09-26

**Authors:** Eleonora Nicolai, Massimo Pieri, Enrico Gratton, Guido Motolese, Sergio Bernardini

**Affiliations:** 1 Department of Experimental Medicine, University of Rome Tor Vergata, Via Montpellier 1, 00133 Rome, Italy; massimo.pieri@uniroma2.it (M.P.); bernards@uniroma2.it (S.B.); 2 Laboratory for Fluorescence Dynamics, Department of Biomedical Engineering, University of California-Irvine, Irvine, CA 92697, USA; egratton22@gmail.com; 3 ASI srl, Via Carroccio 12, 20123 Milan, Italy; gmotolese@yahoo.it; 4 IFCC Emerging Technologies Divison, Via Carlo Farini 81, 20159 Milan, Italy

**Keywords:** antibiotic susceptibility test, urinary tract infection, fluorescence, point-of-care diagnosis, rapid diagnostic testing

## Abstract

Current methods for the diagnosis of urinary tract infections with antimicrobial susceptibility testing take 2–3 days and require a clinical laboratory. The lack of a rapid, point-of-care antibiotic susceptibility test (AST) has contributed to the misuse of antibiotics when treating urinary tract infections (UTIs) and consequently to the rise of multi-drug-resistant organisms. The current clinical approach has led to reduced treatment options and increased costs of diagnosis and therapy. To address this issue, novel diagnostics are needed for the timely determination of antimicrobial susceptibility. We present a rapid, point-of-care, phenotypic AST device that can report the antibiotic susceptibility/resistance of a uropathogen to a panel of antibiotics in as few as 3 h by utilizing fluorescent-labelling chemistry and a highly sensitive particle-counting instrument. We analysed 744 urine samples from the outpatients and inpatients of two Italian hospitals. The 130 UTI-positive patient urine samples we found were measured using a panel of six common UTI antibiotics plus a growth control. By comparing our results to hospital laboratory urine cultures, we obtained an overall sensitivity = 81%, a specificity = 83%, an SPV (sensitivity predicted value) = 95%, and an RPV (resistance predicted value) = 54%. According to our preliminary data, the sensitivity predicted value for a single antibiotic agent was 95%, thus allowing (in the vast majority of cases) an early (within 3 h) recognition of an effective agent for a single patient.

## 1. Introduction

Urinary tract infections (UTIs) are the second leading cause of antibiotic administration, after infections of the respiratory tract. According to EARS (the European Antimicrobial Resistance Surveillance Network), in the 28 countries of the European Union and the two European countries of Iceland and Norway (for a total population of about 500 million inhabitants), the daily consumption rate for the human use of antibiotics exceeds 18.0 doses per thousand people, which corresponds to a daily spread of about 9 million doses, against which the pathogens responsible for the infections are known to develop defense mechanisms [1,2]. According to data released by the WHO (World Health Organization), every year there are 700,000 worldwide deaths from drug resistance caused by the abuse or inappropriate use of antibiotics. Of these, 33,000 are in Europe (with 10,000 in Italy alone) [3,4]. Just under half of the antibiotics taken worldwide are for UTIs. Since the detection of bacterial-origin infections in urine takes, at present, several hours, and the subsequent search for sensitivity to a significant selection of antibiotics extends this time to entire days, very often, in the total absence of information from laboratory tests, broad-spectrum antibiotics are prescribed to patients [5,6]. The antibiotics prescribed could be totally useless, as shown in the negative results from urine cultures (in more than 70% of cases), or they could be ineffective against the pathogen responsible for the infection, as shown by the results of antibiotic susceptibility tests (ASTs) performed in a timely manner [7,8,9]. The possibility of knowing the quantity of the total bacterial load in a urine sample in less than 10 min would greatly limit the need for the prescription of antibiotics for precautionary purposes. Furthermore, the ability to learn the sensitivity of a pathogen present in a sample that tested positive for screening to a selection of antibiotics in only 3 h, although this would be less precise and complete than the results a laboratory would provide after at least two days, would constitute a valuable diagnostic aid for physicians and would be a powerful weapon for healthcare facilities in the battle against drug resistance.

Here, we describe a clinical trial conducted using a new low-cost and easy-to-use technology developed based on a patent from the University of Illinois for urine screening within 8 min [10] and the execution of an AST summary within 3 h. The method used for this clinical trial is based on the well-known liquid in vitro culture technique. Using this technique for screening, the urine samples that are to be analyzed are inoculated into a tube containing a suitable culture broth. An automatic system periodically examines each sample and detects any changes in its optical characteristics. Suitable algorithms transform turbidity variations into the number of living organisms present in the sample. For AST, each sample that tested positive for screening is inoculated into a series of tubes, each of which contains the culture broth and one of the antibiotics to be tested in regard to the pathogen under examination. An additional tube that contains only the sample is used to determine the growth curve of the microorganism in the absence of antibiotics. The tubes are held at a constant temperature of 37 °C and stirred. The sensitivity or resistance of the microorganism to a particular drug is assessed by comparing its growth curve with that detected in the control tube containing the culture broth and the sample without antibiotics. Numerous existing electromedical instruments use this technique for urine screening and for the determination of the resistance of any microorganisms present to a panel of antibiotics selected according to the type of microbe recognized. 

The technique developed and used herein to obtain information on the sensitivity or resistance of the pathogens contained in the samples examined differs from the classical technique described above in two main ways. These changes reduce the analysis time required from two or three days to about 3 h, albeit at the expense of complete information, which must subsequently be obtained from an antibiogram performed by a laboratory.

The first fundamental difference lies in the method used for monitoring the growth of the microorganisms contained in the sample. This is achieved by direct cell counting instead of turbidimetry, leading to a radical reduction in the time needed to obtain the response. In fact, while the measurement of the variation in absorption requires numerous duplications to obtain a meaningful result, the fluorescence count of the number of microbes suspended in a solution, after the appropriate dilution of the sample, is already clearly legible after a few replications using the ASI instrument. This is made possible by the use of a tool described in a US patent obtained by the University of Illinois (US 7,973,294 B2). The instrument uses a new concept for flow cytometry. The device is able to count the fluorescent particles present in a tube, not by making them flow in a thin channel dragged by a conveying liquid, but by making them pass in front of the objective of a reading microscope by moving a disposable cuvette in which a small aliquot of the sample is suspended [11]. The high precision of the instrument, especially at low concentrations [12,13], allows the dilution of urine samples in a 1/100 ratio so that the measurement always takes place in a clear aqueous matrix regardless of the optical characteristics of the whole sample. In the case of the determination of the bacterial population present in a urine sample, given the high concentrations involved (10^4^–10^5^ CFU/mL), the statistics are enriched so rapidly that a period of 30 s is sufficient to obtain reproducible results.

The second crucial difference between the method tested and the one currently used in laboratory analysis lies in the obligation to perform AST without knowing the species of the microbe being examined. At present, there is still no routine method for the rapid recognition of a pathogen present in a sample that tested positive for screening. However, it should be noted that the recognition of pathogens present in urine samples, while essential for a more precise evaluation using AST, and therefore for the execution of a more reliable antibiogram, is perhaps less important, and certainly less urgent, than the rapid determination of antimicrobial susceptibility [14], especially considering that waiting to identify the pathogen would delay the execution of the AST by at least 2 days.

The protocol thus developed firstly consisted of the rapid testing of positive samples, with the total bacterial counts of the sample under examination being determined in less than two minutes [10]; immediately after, we obtained information relating to the sample’s sensitivity to a selected panel of drugs [15]. Having recognized and accepted the limitations deriving from the impossibility of knowing the pathogen under examination, it was decided to proceed assuming that the pathogen belonged to the *Enterobacteriaceae* family, taking advantage of the fact that the species of this family represent at least 80% of all pathogens responsible for urinary tract infections. Under this hypothesis, the antibiotic concentrations of the chosen panel were established using the breakpoint value suggested by EUCAST to discern the sensitivity of the pathogen to each of the antibiotics tested. For those antibiotics for which EUCAST indicated two different values for sensitivity and resistance, we chose to use the value above which the pathogen was not considered to be sensitive, using the principle that it is preferable to have a false resistance indication rather than a false sensitivity indication. For our experimentation, a broad-spectrum panel of antibiotics that could act on the most frequent pathogens found in urinary tract infections was chosen: amoxicillin–clavulanic acid, fosfomycin, and nitrofurantoin for the treatment of uncomplicated UTIs, and ceftazidime, gentamicin, and ciprofloxacin for complicated UTIs, such as relapses or infections related to the use of catheters. For each antibiotic, a test concentration of 10 times the identified breakpoint for concentration-dependent drugs and four times the breakpoint for time-dependent drugs was used (Table 1).

## 2. Results

**Figure 1:**
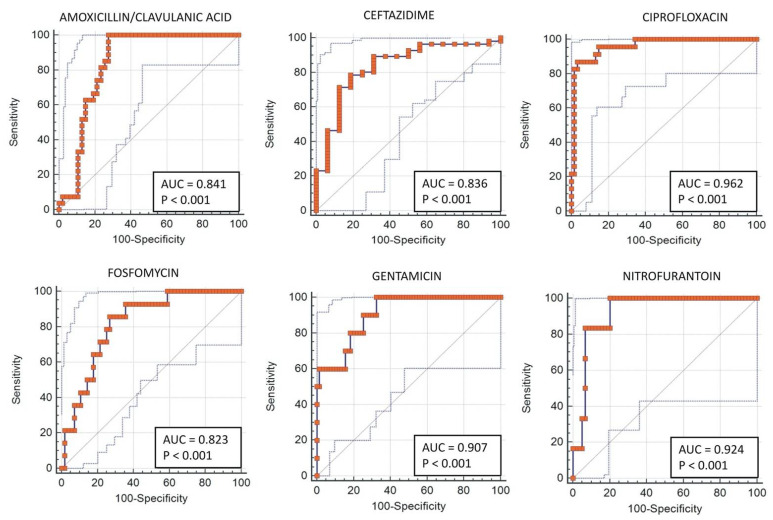
ROC curve obtained for each antibiotic tested.

Using the objective criterion of the ratio between the growth rate of the sample with the antibiotic and that of the control sample, all sample–drug combinations were separated into “sensitivity” or “resistance” cases based on the quantitative growth measurements. Samples with antibiotic/control ratios lower than 0.35 after 3 h were labeled as susceptible (ACR < 35% = “sensitivity”), while those with ratios greater than or equal to 0.35 were labeled as resistant (ACR ≥ 35% = “resistance”). The threshold for the ACR value was calculated using an ROC curve. Figure 1 shows an ROC analysis for each antibiotic tested, with AUC values ranging from 0.836 to 0.962, showing the good discriminating power of the test. Table 2 reports the threshold that optimized the sensitivity and specificity for each antibiotic. The average threshold value, 0.35, was the value chosen to discriminate between sensitivity and resistance in the outcome of the test.

The results obtained for all six antibiotics with regard to the 87 comparable ASTs from the Brotzu and Monserrato hospitals resulted in a sensitivity = 80.7% and a specificity = 83.0% (Table 3). In total, 280/358 susceptible cases and 78/94 resistant cases were correctly identified with respect to the reported clinical AST results (VITEK) for the same samples. Moreover, the sensitivity predicted value (SPV) was 94.6%, and the resistance predicted value (RPV) was 53.8%.

For our test, a low RPV value is not a concern because assigning a wrong resistance value would not lead to a clinical error, only to the choice of different antibiotics. On the other hand, the high SPV value is very important. The ascribing of sensitivity to an antibiotic can determine the prescription of that antibiotic to a patient and is therefore directly linked to clinical practice. We obtained SPV values ranging from 100 to 93.4% for six antibiotics; this value was lower than 90% only for fosfomycin.

**Figure 2:**
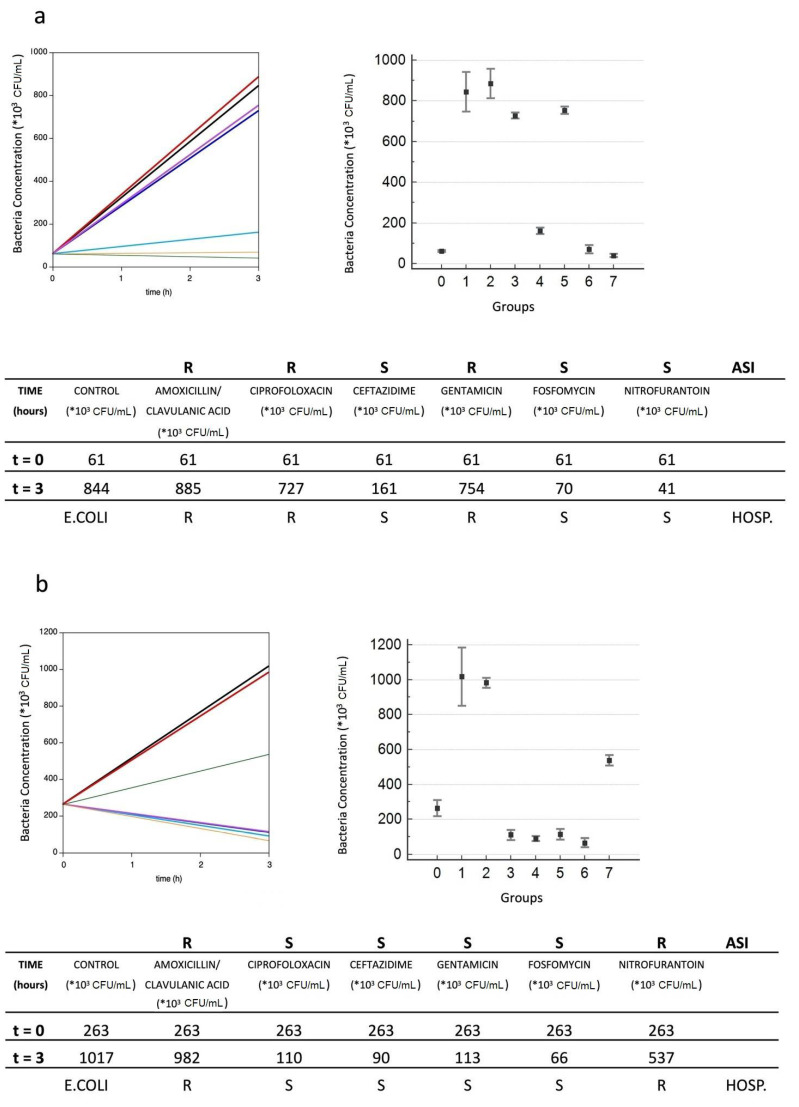

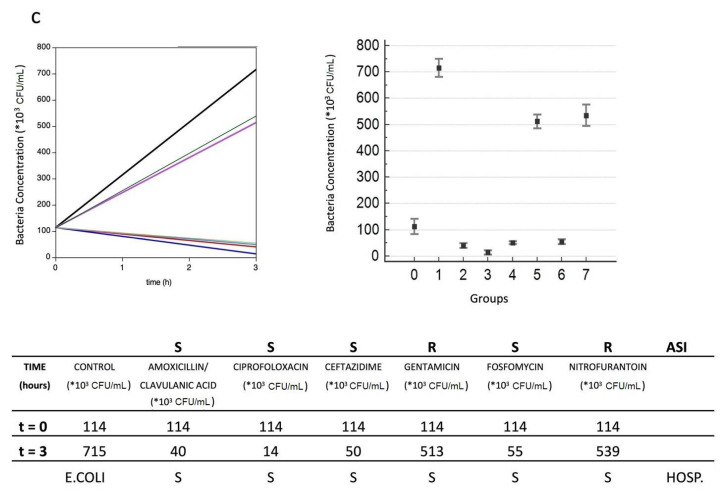
Bacterial concentration versus time for three different samples (**a**–**c**).

In Figure 2, as an example, the results obtained for three samples are reported. The initial and endpoint measurements at 0 and 3 h are plotted. From these graphs, it is clear that the method presented was a rapid and highly sensitive bacterial quantification tool. The response of each sample to a panel of antibiotics is easily related to the identification of the resistant and susceptible bacteria from patient samples. In Figure 2, lines with different slopes are clearly identifiable. Each line corresponds either to samples with different antibiotics or to the control sample. Comparing the slope of the line corresponding to an antibiotic-containing sample to that of control sample permits us to understand whether the pathogen under examination is sensitive or resistant to the respective antibiotic. A line with a slope lower than 0.35 times that of the control refers to an antibiotic to which the pathogen is sensitive; the contrary refers to an antibiotic that the pathogen resists.

An analysis of variance (ANOVA) was performed on the datasets depicted in Figure 2. The distribution of the data was evaluated with the Levene test, which revealed that the data was normal. An ANOVA analysis was therefore performed in combination with a post hoc Bonferroni test, and it was found that *p* < 0.001.

Line graph reports the concentration behavior versus time obtained for the control sample (black line) for samples incubated with amoxicillin–clavulanic acid (red), ciprofloxacin (blue), ceftazidime (light blue), gentamicin (purple), fosfomycin (orange), and nitrofurantoin (green).

Tables report ASI results versus hospital results. The concentrations reported are mean values obtained from three different measurements recorded for each sample. Dot graphs report ANOVA analysis results, where the groups included were: 0 (control at 0 h), 1 (control at 3 h), 2 (sample with amoxicillin–clavulanic acid), 3 (sample with ciprofloxacin), 4 (sample with ceftazidime), 5 (sample with gentamicin), 6 (sample with fosfomycin), and 7 (sample with nitrofurantoin).

## 3. Discussion

Out of a total of 130 antibiograms subjected to the ASI AST test: 87 were positive and comparable to the hospital’s results; 20 were negative and comparable, although they resulted in being positive in the UTI screening both for ASI and for hospitals; and 23 ASI results were not coincident with the hospital results. These 23 noncoincident results are described in detail in Table 4.

From a clinical point of view, for 14 samples out of 23, the clinical prescription would remain the same regardless of the test used. For the nine samples in which the test result was discordant, the ASI test led to the prescription of antibiotics in five cases (3.9% of the total number of samples) in which they would not have been necessary, while it led to the omission of the prescription of antibiotics in the last four cases listed in Table 4 (3.0% of total samples), in which the prescription of antibiotics would have been appropriate. It should be noted that these ASI test errors would be corrected after a few days when the laboratory’s antibiogram became known. 

For the 20 samples that returned negative results from both tests, but which tested positive under screening for both ASI and hospitals, the control did not grow in the antibiogram. One hypothesis that may explain this is that these samples could be RAA (residual antimicrobial activity) positive. For this reason, it will be necessary to include a test for RAA in the future trials, a test not practiced by either of the laboratories involved in this trial. However, these samples, although excluded from the statistics, would have led to the same result from the clinical point of view; that is, the ASI test for these samples would have led to the nonprescription of antibiotics, as would the results of the laboratory test.

## 4. Materials and Methods

### 4.1. The Instrument

**Figure 3:**
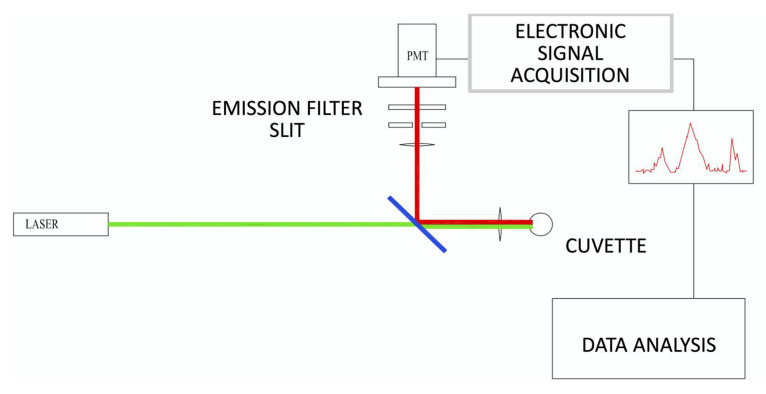
Schematic diagram of the instrument’s components.

The portable, compact, fluorescent particle counter instrument was designed and manufactured by ASI srl (Milan, Italy) for the purpose of identifying fluorescently labelled microbes in liquid media [10]. The instrument consisted primarily of a fluorescence confocal microscope oriented in a horizontal geometry (Figure 3). Briefly, the fast rotation (approximately 5 rev/s) and slower vertical inversion (about 4 mm/s) of the sample cuvette (1 cm in diameter), imparted by two synchronous motors (rotation: Johnson Electric UBR13NB1RN; vertical inversion: Johnson Electric UFR10NB1NR + C.E.M. reducer), resulted in a spiral trajectory that transported labelled bacteria through the optical analysis volume. Excitation light generated by a 532 nm laser diode (Apinex AGLM2-05) with an optical power of less than 3 mW was focused approximately 200 nm within the cuvette by a lens, thereby causing particles in the sample to fluoresce. The emitted fluorescence signal was collected by the same lens and reflected by a dichroic mirror (ODL, SW 570) to the optical sensor through a lens that focused the light on a 200 nm slit in front of a long pass filter (HOYA, 600 nm). The photodetector (PMT Hamamatsu H10721-110) was in optical communication with the confocal microscope and received a portion of the fluorescence from the observation volume, measuring its intensity as a function of time and generating a temporal profile of the fluorescence from the observation volume. The electric signal output by the PMT was amplified and digitized at 40 kHz and transmitted to a PC. In real time, the processor applied a pattern recognition algorithm to the temporal profile to determine the concentration of particles in the sample. The algorithm matched features in the temporal profile to predetermined patterns that corresponded to the time-dependent fluorescence intensity of particles passing through the observation volume. The concentration of particles was determined by calculating the number of predetermined patterns matched to features in the temporal profile for a given sample scanning period. Concentrations were extracted from the analyzed temporal profile by dividing the number of matches by the volume of the sample analyzed during a selected sample scanning period.

### 4.2. Fluorescent Probe

The chosen fluorescent probe was SYTOX ORANGE (Thermo Fisher Scientific, Waltham, MA, USA), a DNA marker that does not penetrate the walls of living microorganisms. Therefore, to carry out the measurement, it was necessary to damage the microbe wall by heating the measuring cuvette at 80 °C for 7 min. 

### 4.3. Patient Enrollment

A total of 744 urine specimens were collected from both inpatients and outpatients from Brotzu Hospital (*n* = 603) (Cagliari, Italy) and Monserrato University Hospital (*n* = 141) (Cagliari, Italy) over a period between June 2018 and October 2018 with informed consent from donors and approval from the University Hospital of Cagliari Institutional Review Board (PG/2018/5211). Each sample was identified by a serial code with no patient information, such as sex or age. Prior to inclusion in the AST pilot evaluation, urine samples, collected as part of routine clinical evaluation by the microbiology laboratories of each hospital, were screened for “presumptive UTI positive” status.

UTI status was investigated by the Monserrato laboratory by plating all samples, by the Brotzu laboratory using Alfred (Alifax), and using the particle counter system by ASI (ASI, Milan, Italy), as described previously [10]. Reported concentrations were recorded in CFU/mL. Because of personnel limitations, a maximum number of four presumptive positive samples per day were enrolled in the AST pilot evaluation (*n* = 1–4 samples per day). A total of 130 samples were positive at the screening. Of these, 44 ASTs were performed at Monserrato University Hospital and 86 were performed at Brotzu Hospital. All samples were examined within 5 h of receipt in the laboratory. The AST results obtained by ASI within 3 h were compared with the results of the two hospital laboratories when the latter became available.

### 4.4. Clinical Antibiotic Sensitivity Testing Using Conventional Systems

Clinical antibiotic sensitivity tests were performed at each hospital laboratory using the automated system VITEK^®^2 (BioMérieux, Marcy-l’Étoile, France). 

Presumptive and species identification of bacteria was first performed using the LinearCount6^®^ test and VITEK^®^ MS. Then, a standardized inoculum at 0.5 McFarland turbidity was prepared in 0.9% saline solution using VITEK^®^2 DensiChek. Antibiotic sensitivity tests were performed using single-use AST-N376 cards for Gram-negative bacteria, AST-N659 for *Staphylococcus* spp., and AST-N658 for *Enterococcus* spp. The reading times varied from 5 (threshold value) to 11 h for slow-growing microorganisms. 

### 4.5. Clinical AST Analysis with Rapid Bacterial Quantification System

A panel of six commonly used UTI antibiotics (amoxicillin–clavulanic acid (AX), ciprofloxacin (CP), ceftazidime (CZ), gentamycin (GM), fosfomycin (FF), and nitrofurantoin (NF)) was selected, with concentrations defined based on the MIC thresholds outlined by the European Committee on Antimicrobial Susceptibility Testing (EUCAST) guidelines [16]. Specifically, 4× maximum MIC concentrations were used for time-dependent drugs (AX, CZ, NF), and 10× maximum MIC concentrations were used for concentration-dependent drugs (CP, GM, FF) [17,18,19].

For each patient sample, seven tubes containing 3 mL of broth (LB, Sigma-Aldrich^®^, St. Louis, MO, USA) were prepared, one for each of the antibiotics in the panel, as well as a positive growth control without antibiotics. For each unique patient sample that tested positive during UTI screening, based on sample concentrations obtained during UTI screening, an aliquot of the raw urine sample was spiked into each of the seven culture tubes for a final concentration of 5 × 10^4^ CFU/mL. The seven cultures were incubated at 37 °C without stirring.

At the timepoints of 0, 1, 2, and 3 h, 30 μL from each tube was collected and added to a cuvette containing 3 mL of isotonic solution plus 30 μL of Sytox Orange 50 mM, heated for 7 min at 80 °C and scanned for 30 s on the particle counter unit. All culture tubes were incubated at 37 °C between subsequent time points. 

### 4.6. Data Analysis

For each sample, the growth curve of the culture containing the antibiotic was compared with the growth curve of the control tube containing no drugs. The microorganisms contained in the tubes in which the growth did not exceed 35% of the growth in the reference tube were considered sensitive to the corresponding antibiotics.

### 4.7. Statistical Analysis

Descriptive statistics, such as mean and standard deviation (SD), were calculated. The normality of all data was determined by Leneve normality test. In the case of a normal distribution, parametric tests were used, such as ANOVA with a Bonferroni post hoc test. Receiver operating characteristic (ROC) curve analysis was used to determine the threshold for ACR values. A *p*-value lower than 0.05 (*p*-value < 0.05) was considered statistically significant. All analyses were performed using Med Calc Ver. 18.2.18 (MedCalcSoftware Ltd, Ostend, Belgium).

## 5. Conclusions

The AST technologies available today cannot provide physicians with antibiotic susceptibility results in time to inform early treatment decisions. The instrument and protocol described in this study represent a prototype point-of-care device to perform rapid phenotypic UTI AST in a clinical evaluation.

In addition to not requiring the enrichment of bacteria from urine specimens, our AST device does not require any preprocessing steps, such as centrifugation or wash steps, simplifying the necessary instrumentation for point-of-care applications. Furthermore, our instrument can directly detect microorganisms at the single-cell level, eliminating the need for CFU plating, which has a time-to-answer of at least 24 h before obtaining visible bacterial growth. Unlike culture-based AST, our device provides objective, quantitative information to the end user, and is not vulnerable to inaccurate or subjective interpretations due to variables, such as user-dependent differences in sample preparations, plating techniques, and zone-diameter measurements [20].

## Figures and Tables

**Table 1:** Antibiotic concentrations used.

Eucast	Enterobacteriaceae		
	MIC Breakpoint (mg/L)		Tube Concentration (mg/L)
	S≤	R>	
Amoxicillin– Clavulanic Acid	32	32	128
Ceftazidime	1	4	4
Ciprofloxacin	0.25	0.5	2.5
Gentamicin	2	4	20
Fosfomycin	32	32	320
Nitrofurantoin	64	64	256

**Table 2:** Cutoff values for optimal sensitivity and specificity values obtained by the ROC curve analysis.

Antibiotic	Cutoff	Sensitivity	Specificity
Amoxicillin–Clavulanic Acid	0.41	100.00	72.34
Ceftazidime	0.63	78.57	81.25
Ciprofloxacin	0.47	86.96	96.72
Fosfomycin	0.10	85.71	73.21
Gentamicin	0.19	100.00	67.61
Nitrofurantoin	0.31	100.00	79.66

**Table 3:** Statistical parameters calculated for all datasets and for each single antibiotic tested.

Total			Amoxicillin/Clavulanic Acid			Ciprofloxacin			Ceftazidime		
	S_hosp_	R_hosp_		S_hosp_	R_hosp_		S_hosp_	R_hosp_		S_hosp_	R_hosp_
S_ASI_	280	16	S_ASI_	33	0	S_ASI_	56	3	S_ASI_	31	2
R_ASI_	67	78	R_ASI_	14	27	R_ASI_	5	21	R_ASI_	25	14
Sensitivity = 80.7% Specificity = 83.0% SPV = 94.6% RPV = 53.8%			Sensitivity = 70.2% Specificity = 100% SPV = 100% RPV = 65.8%			Sensitivity = 91.8% Specificity = 87.5% SPV = 94.9% RPV = 80.8%			Sensitivity = 55.3% Specificity = 87.5% SPV = 93.9% RPV = 35.9%		
			**Gentamicin**			**Fosfomycin**			**Nitrofurantoin**		
				S_hosp_	R_hosp_		S_hosp_	R_hosp_		S_hosp_	R_hosp_
			S_ASI_	61	3	S_ASI_	52	8	S_ASI_	47	0
			R_ASI_	8	8	R_ASI_	4	7	R_ASI_	11	1
			Sensitivity = 88.4% Specificity = 72.7% SPV = 95.3% RPV = 50.0%			Sensitivity = 92.9% Specificity = 46.7% SPV = 86.7% RPV = 63.6%			Sensitivity = 81.0% Specificity = 100% SPV = 100% RPV = 8.3%		

Notes: SPV = TS/(TS + FS) = (sensitivity predicted value); RPV = TR/(TR + FR) = (resistance predicted value); sensitivity = TS/(TS + FR); specificity=TR/(TR + FS).

**Table 4:** Discussion of samples for which the test results were not in accordance with the hospital laboratory results.

Sample	Hospital	ASI	Clinical Outcome Hospital	Clinical Outcome ASI
8	Candida	In 5, the control did not grow; In 3, all antibiotics tested were found resistant.	No antibiotic prescription.	No antibiotic prescription.
11	MMF (mixed microbial flora)	In 4, the control did not grow; In 2, all antibiotics tested were found resistant; In 5, both resistant and sensitive values were given.	No antibiotic prescription (in case of MFF results, hospital doesn’t perform AST).	For 6, no antibiotic prescription; For 5, antibiotic sensitivity was suggested.
4	Two positives for single species: -ps. Aeruginosa -prot. Mirabilis 2 positives for double species: -prot. Mirabilis + ent.co faecalis -e.coli + ent. Faecalis	In 4, control did not grow.	Yes antibiotic prescription.	No antibiotic prescription.

## Data Availability

Data sharing not applicable.
